# ‘When I had concerns about my own patients…I was told to keep quiet’: experiences of moral injury among healthcare workers under Covid-19 vaccine mandates in Alberta, Canada

**DOI:** 10.1515/med-2026-1503

**Published:** 2026-08-18

**Authors:** Claudia Chaufan, Natalie Hemsing, Rachael Moncrieffe

**Affiliations:** School of Health Policy and Management, York University, Toronto, Canada

**Keywords:** Covid-19 vaccine mandates, healthcare workers, moral injury, health governance, manual sentiment analysis

## Abstract

**Objectives:**

Although compliance is often interpreted as consensus and dissent as “vaccine hesitancy”, misinformation, or deficient trust, less is known about how healthcare workers experienced Covid-19 vaccine mandates or how their legitimacy was constructed and contested.

**Methods:**

We analyzed open-ended survey responses from 80 respondents within a convenience sample of 189 healthcare workers in Alberta, Canada. Informed by Weberian ideal types, responses were classified by normative orientation toward mandates and analyzed thematically.

**Results:**

Most respondents (82.5 %) expressed negative sentiments, while a minority (17.5 %) were positive; none were neutral. Negative accounts emphasized coercion, institutional betrayal, silencing, ethical conflict, and moral injury, including among vaccinated respondents. Positive responses emphasized patient care, role expectations, and public safety, often framing dissent as illegitimate.

**Conclusions:**

The findings indicate that mandates were experienced as professionally divisive and, by many respondents, as ethically disruptive; that compliance should not be equated with ethical alignment; and that high vaccination uptake can obscure coercive conditions. Treating dissent as an empirical object reveals how mandate legitimacy was constructed and how institutional practices managed disagreement. Moral injury emerged not merely as an individual psychological response, but as a structurally induced ethical harm with implications for professional judgment, informed consent, patient care, workforce stability, and institutional integrity.

## Introduction

Covid-19 vaccination mandates for healthcare workers were adopted across many jurisdictions as employment-based policies, justified in the name of patient safety, workforce protection, and healthcare system capacity [1]. In Canada, as elsewhere, these mandates were introduced in a period of rapid policy change, contested science, and unresolved ethical conflict. While supporters framed mandated vaccination as a necessary expression of professional duty and collective responsibility, many healthcare workers experienced these policies as coercive, ethically troubling, and professionally destabilizing [2], 3].

Alberta’s Covid-19 response provides a particularly salient case for examining these tensions. Compared with most Canadian provinces, Alberta was marked by lower vaccine uptake, pronounced political polarization, and public conflict over the legitimacy of mandated vaccination. Early in the vaccine rollout, Alberta had among the highest reported levels of vaccine refusal in Canada, and by late 2021 its vaccination rate remained well below the national average [4], 5]. Against this background, Premier Jason Kenney announced a province-wide restrictions exemption program in September 2021, after months of rejecting the premise of a vaccine passport [6]. Soon afterward, Alberta Health Services – the province’s single health authority – introduced a vaccination mandate for healthcare workers, contractors, and other health-sector personnel, requiring full vaccination as a condition of employment [7].

The policy was defended by institutional leaders and some healthcare providers and unions as necessary to ensure safety in healthcare settings [8], [9], [10]. It also provoked opposition from other healthcare workers, public protests, and legal challenges [9], 11]. Alberta Health Services later delayed the mandate, in light of staffing shortages, particularly in rural and already underserved areas [12], and subsequently modified it to allow unvaccinated workers to return under a testing regime [13]. By February 2022, Kenney publicly questioned its defensibility, stating that vaccinated healthcare workers were “pretty much as likely to transmit as unvaccinated” and that, given workforce challenges, the policy was “no longer defensible” [14]. In July 2022, Alberta Health Services rescinded the mandate, even as it continued encouraging healthcare workers and all Albertans to “review current evidence from reputable sources to make informed decisions about getting immunized”, thereby implying continuing support for the practice [15]. These reversals provide an important policy backdrop for examining how healthcare workers understood and experienced the mandate as it unfolded.

Much of the literature on healthcare workers and Covid-19 vaccination has been organized around the category of “vaccine hesitancy”, used to describe delay, uncertainty, or refusal despite vaccine availability, and identified by the World Health Organization as one of “10 threats to global health”, alongside famine, weak primary health care, and antimicrobial resistance [16]. Although often presented as a neutral descriptor, this framing has important analytic and political effects. It locates the problem primarily in healthcare workers’ attitudes, emotions, knowledge deficits, mistrust, or susceptibility to “misinformation”, rather than in the evidentiary, ethical, institutional, or coercive features of the policies themselves. As we argued in prior work, this framing pathologizes dissent, converting disagreement, caution, or demands for evidence into problems of cognition, trust, communication, or behaviour to be corrected [17].

Recent studies illustrate this pattern. Carpenter et al. [18] found that nurses who declined Covid-19 vaccination described being pressured, devalued, divided from colleagues, and mistreated or discriminated against [18]. Christianson et al. [19] similarly identified coercion, perceived immunity, concerns about pre-existing health conditions, and mistrust of official claims among US nurses, while also noting that vaccine mandates may worsen workforce attrition [19]. Ledda et al. [20] examined 52 Italian healthcare personnel suspended for refusing Covid-19 vaccination and found high knowledge of recommended occupational vaccines alongside near-universal opposition to mandatory vaccination policies, mistrust of authorities, and concerns about vaccine safety and benefits [20]. Kung et al. [2], in a systematic review of ethical challenges around mandatory vaccination among nurses, identified stigma, moral distress, workplace division, safety concerns, and distrust as recurring themes [2].

This literature is valuable because it documents ethical distress, workplace harms, stigma, and loss of trust, and recognizes that moral injury or moral distress can arise from organizational and system-level conditions. However, it also illustrates the limits of a frame that centres the “hesitant”, resistant, or non-compliant healthcare worker as the primary analytic object. Even when these studies may sympathetically describe coercion, marginalization, and professional disruption, they often continue to treat resistance as a problem to be explained, mitigated, or overcome through better information, education, communication, counselling, trust-building, leadership, or emotional support. In some cases, refusal is explicitly attributed to “anti-vaccination beliefs” or “culture” [20], while the corresponding pro-vaccination orientation is framed in affirmative terms, such as “safety culture”, professional responsibility, or collective protection [2]. This asymmetry matters because it leaves the policy assumptions that generated these distressful experiences subject to far less scrutiny.

A related limitation appears in ethical analyses that frame mandates primarily as a conflict between individual autonomy and collective welfare [21]. This framing is incomplete because it assumes what must first be demonstrated: that the public good required employment-based vaccination mandates for healthcare workers. More specifically, appeals to collective welfare do not, on their own, establish that a specific restrictive policy was necessary, proportionate, evidence-informed, or consistent with bioethical principles. This distinction matters because healthcare workers had provided care throughout 2020 and much of 2021 – they were even framed as “heroes” in academic and lay discourse [22], 23] – before mandates were introduced and even before vaccines were available. Their later reclassification as unacceptable risks to patients or to system capacity therefore requires empirical justification that they posed such risk [24]. In this sense, treating mandate legitimacy as the neutral starting point is itself a normative position, rather than a conclusion established by the evidence.

The claim of mandate legitimacy, however, was not empirically self-evident. Although public health authorities and institutional leaders presented employment-based vaccination mandates as necessary to protect patients and preserve healthcare-system capacity, by the time mandates were imposed, the evidentiary landscape was already complex. Early estimates of Covid-19 infection fatality rates indicated substantial age stratification and relatively low risk among non-elderly populations [25], 26]. Evidence of naturally acquired immunity was also well established [27], including among healthcare workers: the SIREN study, a large multicentre prospective cohort study of more than 25,000 healthcare workers in England, found that previous SARS-CoV-2 infection was associated with substantially reduced risk of reinfection for at least several months, independent of vaccination [28]. Concerns had also been raised that pivotal vaccine trials were not designed to determine effects on transmission, hospitalization, or mortality, and that relative risk reduction was emphasized to the detriment of absolute risk approaches [29], 30] – the recommended type for risk communication [31].

Subsequent studies documented breakthrough infections and transmission among vaccinated persons, including healthcare workers and patients in healthcare settings [32], [33], [34], [35]. Other analyses found no clear relationship between vaccination rates and case incidence across multiple jurisdictions during the Delta period [36]. Safety concerns and adverse-event signals were also identified early in the peer-reviewed literature – including serious adverse events of special interest, myocarditis, and other post-vaccination outcomes requiring careful risk-benefit assessment [37], [38], [39] – and have continued to be documented in subsequent studies [40], 41].

Adjudicating the full biomedical literature on Covid-19 vaccination is beyond the scope of this qualitative study. However, the contested biomedical and policy context is directly relevant to the analysis, because healthcare workers’ accounts of coercion, exclusion, silencing, and moral injury did not arise in a vacuum. They emerged in a policy environment in which vaccination status was treated not merely as a clinical or administrative category, but as a marker of professional responsibility, patient safety, and moral worth. Employment-based mandates were therefore not simply neutral applications of scientific fact, but restrictive workplace policies grounded in particular interpretations of evidence, risk, professional duty, and institutional authority. For healthcare workers, these policies were embedded in patient-care obligations, disciplinary expectations, and institutional messaging that often required them not only to comply, but also to enact, defend, or remain silent about policies they considered ethically or scientifically questionable.

The concept of moral injury is useful for analyzing healthcare workers’ experiences. In the health services literature, moral injury has been defined as the distress that arises from participating in, witnessing, or being unable to prevent actions that violate deeply held ethical principles [42], 43]. As a distinct form of moral harm, it offers a useful lens through which to interpret many responses in our dataset. The term itself originated in the context of war psychiatry, where it was introduced by Jonathan Shay to describe the long-term psychological and character harm experienced by combat veterans – particularly those of the Vietnam War – following a perceived betrayal of what is right by someone in a position of authority in a high-stakes situation.

Shay identified this pattern both in clinical encounters and in Homeric literature, treating the Iliad and Odyssey as enduring experiments in the ethics of military leadership and cohesion [43], 44]. In the Covid-19 period, the concept was applied to healthcare workers facing extreme workloads, triage dilemmas, resource shortages, and institutional constraints [45]. In this study, however, we approach moral injury not primarily as a clinical outcome located within the psychology or emotions of individual workers, but as a structural and institutional phenomenon: an ethical rupture produced when workers perceive that they are required to comply with, enforce, or remain silent about policies that conflict with professional or personal ethics, informed consent, patient care, or evidentiary integrity.

This study examines the lived experiences of healthcare workers in Alberta who were subject to Covid-19 vaccination mandates as a condition of employment. Drawing on manual qualitative sentiment analysis of responses to one open-ended survey question completed by 80 respondents within a convenience sample of 189 healthcare workers, we analyze positive, negative, and neutral orientations toward the policy, with particular attention to accounts of coercion, professional silencing, ethical conflict, and moral injury. Informed by the quantitative findings from the broader survey, the study treats dissent not as a deficit to be explained or corrected, but as an empirical object of inquiry. It therefore examines both how healthcare workers experienced and interpreted the mandates and how mandate legitimacy, professional authority, and acceptable dissent were constructed and contested in practice. The study is part of a broader research program examining Covid-19 workplace policies among healthcare workers across Canadian provinces.

## Materials and methods

We conducted a qualitative, manually coded sentiment analysis of responses to a single open-ended question (“Are there any other issues that you believe would help us to further understand the impact of vaccine mandates on healthcare workers and patient care? If so, please elaborate”), drawing on responses from 80 participants within a broader survey of 189 healthcare workers recruited through convenience sampling. Quantitative results from the full survey have been reported separately [46]. Respondents were eligible if they had worked in Alberta’s healthcare sector during the mandate period – whether in clinical, administrative, or support roles – and regardless of whether they were still employed at the time of the survey or had been laid off, taken early retirement, or otherwise exited the workforce due to mandate-related pressures.

The initial survey, delivered through Google Forms, was promoted through social media platforms and professional networks of the research team using a snowball sampling approach [47] that encouraged participants to share the invitation with other eligible healthcare workers. Healthcare workers across Alberta were invited, regardless of vaccination status, profession, age, gender, years of experience, socioeconomic background, or race/ethnicity. Recruitment took place over March and April 2025, with invitations redistributed at seven-day intervals [48].

Potential participants were extended an information letter including details on study aims, methods, potential benefits and risks, and confidentiality. They were also informed of their right to withdraw consent at any time without consequences. The online survey questions were only accessible to participants after they provided their freely informed consent. The study was approved by the York University Office of Research Ethics (no. 2023-389).

The sentiment analysis approach, defined as the study of “opinions, sentiments, evaluations, attitudes, and emotions as expressed in natural language text” [49], was conducted through manual typological and thematic classification rather than computational text mining. This interpretive approach to sentiment coding – consistent with scholarship emphasizing the importance of human over machine interpretation in capturing nuanced meaning – [50], 51] enabled us to identify broader thematic patterns while remaining attentive to heterogeneity within each sentiment category. In practice, responses were categorized according to their evaluative stance toward Covid-19 vaccine mandates, whether explicit or implicit, and classified as positive, neutral, or negative. These categories were conceptualized not as mere emotional valences, but as Weberian ideal types – heuristic constructs representing distinct normative orientations – [52] and were used to assess each response based on its proximity to one of the types, reflecting the respondent’s ethical stance toward mandated Covid-19 vaccination policy.

The analysis proceeded in five stages. First, all authors read the full dataset of qualitative entries. Second, coding procedures were piloted on a small subset of responses to refine interpretive consistency across sentiment categories. Entries were classified through close reading to identify their dominant evaluative orientation, taking into account both explicit statements and implied judgments conveyed through tone, narrative framing, and ethical positioning. Classification was based on both content and tone.

Positive entries explicitly supported vaccine mandates, criticized those who resisted them, or affirmed institutional policies with little or no qualification. Negative entries included any form of critique, skepticism, or distress related to the mandates, such as expressions of coercion, regret, ethical tension, professional harm, loss of trust in institutions, or concern for the treatment of unvaccinated colleagues. Responses by vaccinated participants who emphasized informed choice or criticized the marginalization of dissenting healthcare workers were also categorized as negative with respect to mandate policy, as they expressed disapproval of the mandates despite having complied. Where responses contained mixed or nuanced positions, classification was based on the predominant evaluative stance expressed in the entry as a whole. The neutral category was reserved for responses that lacked a clear evaluative position (Table 1).

**Table 1:** Sentiment categorization criteria.

Sentiment category	Criteria
Negative	– Opposes vaccine mandates even if vaccinated – Laments unjust treatment of unvaccinated colleagues – Expresses regret, pressure, coercion, or institutional failure – Advocates for freedom of choice or critiques mandate implementation
Positive	– Explicitly supports both vaccination and mandates – Criticizes those who resisted mandates
Neutral	– Factual, vague, or lacks evaluative judgment about mandate supporters or dissidents

Third, two authors independently coded the full set of responses and compared their classifications. Disagreements were infrequent and were resolved through discussion and re-examination of the textual evidence until consensus was reached. Fourth, after applying this categorization, we thematically analyzed entries [53] within each sentiment group to identify recurring concepts, experiences, and narrative patterns. Fifth, selected entries were included in the results section when appropriate to support our analysis, retaining their original survey identification number.

At this point, it is worth noting that while moral injury was not part of the study design – no survey question explicitly asked about it – the survey deliberately allowed respondents to report negative experiences and dissent without framing them as problematic or funneling them through subtly normative concepts such as “vaccine hesitancy,” “misinformation,” and the like. As suggested in the Introduction, this design may have allowed respondents, particularly in the open-ended question, to articulate experiences through which moral injury became analytically visible, in turn enabling us to identify it inductively as we read the open-ended entries.

### Statement on reflexivity

The research team consisted of three female investigators with over four decades of combined experience in medical practice, medical sociology and anthropology, bioethical analysis, and health policy. In addition to our academic and professional expertise, our engagement with the topic of vaccine mandates stems from longstanding concerns about the impact of Covid-19 policies on the health and postsecondary sectors in Canada. These concerns are reflected in a substantial body of individual and collaborative research [54], [55], [56], [57], [58]. Following Malterud [59], these disclosures are offered as a means of enhancing transparency and to ground our commitment to epistemic integrity.

## Results

Among the 80 healthcare workers who responded to the open-ended survey question, 66 (82.5 %) expressed negative sentiments regarding Covid-19 vaccines or mandates. All unvaccinated respondents (41/41; 100 %) and all partially vaccinated respondents (4/4; 100 %) expressed negative sentiments. Among fully vaccinated respondents (15/80; 18.8 %), 14 (93.3 %) expressed negative sentiments and one (6.7 %) expressed a positive sentiment. Sentiment among boosted respondents was more mixed: all respondents boosted once (6/6; 100 %) expressed positive sentiments, whereas among those boosted twice or more (14/80; 17.5 %), seven (50 %) expressed positive sentiments and seven (50 %) expressed negative sentiments. While most responses closely aligned with these ideal types, a small number were more ambiguous; however, closer examination indicated that all entries reflected an evaluative orientation either in support of or against mandated Covid vaccination. No response was coded as “neutral”, as none fit the description of the category, indicating a pronounced polarization across respondents (Table 2).

**Table 2:** Sentiment analysis distribution.

Vaccination status	Number of respondents, n	Percentage of total, %	Sentiment distribution
Unvaccinated	41	51.3 % (41/80)	100 % (41/41) negative
Partially vaccinated	4	5.0 % (4/80)	100 % (4/4) negative
Fully vaccinated	15	18.8 % (15/80)	93.3 % (14/15) negative
			6.7 % (1/15) positive
Boosted once	6	7.5 % (6/80)	100 % (6/6) positive
Boosted twice or more	14	17.5 % (14/80)	50 % (7/14) negative
			50 % (7/14) positive

Table 3 summarizes the key themes identified within each normative orientation. These themes are elaborated below and illustrated with selected respondent quotations.

**Table 3:** Key themes within normative orientations.

Normative orientation	Key themes
Negative sentiment toward mandates	– Coercion, distress, and ethical conflict – Bodily autonomy and medical ethics – Institutional betrayal and loss of trust – Suppression, silencing, and retaliation – Solidarity with marginalized colleagues – Moral injury and professional dislocation
Positive sentiment toward mandates	– Professional duty and public health responsibility – Vaccination as patient protection – Frustration with unvaccinated colleagues – Vaccination status as professional legitimacy – Critical inquiry as bias or illegitimate dissent
Neutral sentiment toward mandates	– No neutral responses identified

### Negative sentiment toward mandates

Of the 80 respondents, a substantial majority – 66 entries (82.5 %) – expressed negative sentiments toward Covid-19 vaccine mandates. Notably, these responses came not only from unvaccinated and partially vaccinated participants, but also from 14 of 15 fully vaccinated respondents and 7 of the 14 who were boosted twice or more times. This distribution indicates that compliance with mandates did not necessarily equate endorsement and that opposition to these policies extended across vaccination statuses.

Several themes were identified consistently across these accounts, with respondents describing vaccine mandates as *psychologically coercive, emotionally distressing, and ethically troubling*. Many spoke of being forced to choose between their professional identities and deeply held moral or bodily integrity. One fully vaccinated respondent wrote: “I felt completely manipulated and toyed with… I suffer from PTSD and depression from the mandates and subsequent vaccination” (Entry 96). Another recalled complying “at the very last opportunity”, expressing abandonment by both employer and union (Entry 64). These accounts reflect the emotional and psychological toll of navigating high-stakes compliance under threat of exclusion.

Closely linked to these accounts was a pronounced sense of *violation of bodily autonomy and principles of medical ethics*. Multiple respondents described the mandates as antithetical to the values that healthcare is meant to uphold. An unvaccinated nurse described the policy as “inhumane and unethical… a breach of patient confidentiality, informed consent and bodily autonomy” (Entry 133). Another, who had received the vaccine, criticized what they viewed as ethical inconsistency: “We were hypocritical with our medical ethics… We tell a woman she has a right to abortion… but that same woman should then have no choice in taking a vaccine” (Entry 143). These statements reflect an internalization of professional codes of ethics and a belief that those codes had been violated by institutional policy.

Participants across vaccination statuses expressed a pronounced sense of *institutional betrayal and erosion of trust* in professional bodies, regulatory colleges, and unions. This theme appeared across most vaccination statuses but was especially pronounced among those who had been dismissed or denied accommodations. A partially vaccinated respondent reflected: “The forcefulness of mandating vaccines removed the choice on own body by losing your job security and has led to many believing that they can no longer trust doctors or government” (Entry 177). An unvaccinated worker described their treatment as “the worst human experience I’ve ever been through” (Entry 90), illustrating the moral weight participants attributed to what they experienced as punishment, humiliation, and abandonment. These narratives were also marked by *reports of suppression and retaliation*. For example, several described being told not to report vaccine-related injuries or being disciplined for raising concerns. One noted simply: “Active discouragement of reporting vaccine side effects” (Entry 180). Others described workplaces where conversation was tightly controlled and dissent was punished: “Any discussion around vaccination status or adverse reactions resulted in disciplinary action” (Entry 181).

Importantly, not all negative responses came from those who had refused vaccination. Indeed, several vaccinated respondents expressed *solidarity with colleagues who had been marginalized, discriminated against, or dismissed.* One healthcare worker who had ultimately complied, expressed regret and described ethical tension: “There was a significant lack of support for healthcare professionals that chose not be vaccinated” (Entry 94). These expressions reveal a broader undercurrent of professional and moral concern and complicate assumptions that having accepted vaccination equates normative alignment with the policy. Taken together, these reflections constitute a coherent critique of the mandates as unjust, harmful, and at odds with professional ethics and democratic norms. They also underscore that the boundary between compliance and dissent was not as clear-cut as institutional policies presumed: many respondents complied, but under protest – and with lasting emotional and moral consequences.

The most salient theme within the broader pattern of negative sentiment was the *rupture in moral orientation*, namely, experiences of moral injury, evident in a number of entries from across vaccination statuses. Several participants described being forced to act against their professional or ethical convictions, to remain silent in the face of perceived wrongdoing, or to watch patients or colleagues suffer avoidable harm while feeling powerless to intervene. For example, one fully vaccinated respondent wrote: “I was forced to inject an experimental substance or lose the ability to provide for my family… I wish I had been stronger and refused” (Entry 64), reflecting what the respondent described as the lasting psychological consequences of ethical compromise.

Another respondent, whose comments form the basis of the title of this article, recalled: “When I had concerns about my own patients’ negative reactions… I was told to keep quiet” (Entry 53), while a third reported, “The guilt rots away at my head” (Entry 90), a phrase that conveys the lingering emotional and moral impact of these experiences. Another respondent, reflecting on what they perceived as shifting institutional standards, wrote: “Initially, the vaccines were not approved for pregnant women… then all of a sudden, we were told to vaccinate pregnant women… When I asked for the evidence, I was told there was NONE, and it wasn’t my job to ask questions… I left my job not long after that” (Entry 99). These accounts and others like them point not only to dissent or discomfort but to a deeper and more enduring rupture: the experience of institutional betrayal and moral dislocation.

### Positive sentiment toward mandates

A small minority – 14 of 80 respondents (17.5 %) – expressed positive sentiments toward Covid-19 vaccine mandates. All of these responses came from those who were boosted either once, twice or more times, with the exception of a single fully vaccinated respondent, who also expressed positive sentiments. These participants framed the mandates not as coercive or ethically troubling, but rather as scientifically justified, professionally responsible, and morally imperative. The most prevalent theme among these responses was a strong sense of *professional duty and alignment with public health ethics*. For these participants, vaccination – and requiring others to be vaccinated – was consistent with the ethical obligations of healthcare work.

For example, one respondent emphasized: “I was happy to get vaccinated to protect the vulnerable populations I was working with” (Entry 89), affirming both personal compliance and moral commitment to what they described as professionally responsible and protective of the broader community. Another elaborated, “As healthcare workers, we are required to show vaccine status… I strongly feel we should support the health of the greater community” (Entry 31). These sentiments reflected not just agreement with mandates and their assumed scientific underpinnings, but the belief that mandates reaffirmed the fundamental norms of healthcare practice and professional ethics.

Another recurring theme was *frustration with unvaccinated colleagues*, who were often portrayed as undermining professional integrity. Some respondents expressed concern or disdain for what they perceived as recklessness towards patients, or irrational fear of Covid vaccines among some colleagues. One declared: “Those [healthcare workers] who elected not to get immunized… had an impact on me. The lack of accountability as a professional to keep patients safe was deplorable” (Entry 46). Another stated unequivocally: “[healthcare workers] who refused to be vaccinated ultimately put their patients at risk which is ethically wrong” (Entry 39). These critiques suggest that for this group, vaccination status functioned as a marker of professional legitimacy and moral integrity.

A subset of healthcare workers who expressed positive sentiments toward vaccine mandates went further by explicitly *challenging the legitimacy of the study.* Rather than merely endorsing mandates, these respondents objected to the inclusion of survey statements that allowed respondents both to report negative experiences, including vaccine-related harms, and evaluate critical perspectives on mandate enforcement, suggesting that the availability of such reporting and evaluation was itself experienced as inappropriate or biased. One respondent asserted that “these questions have implicit bias that assumes negative experiences” (Entry 59), while another expressed concern about the study’s underlying rationale, stating: “I actually think this survey is biased towards supporting an assertion that the policies were more harmful than beneficial to workers” (Entry 128).

Taken together, these responses indicate that for this subset of mandate supporters, the very act of asking about adverse experiences and ethical concerns was interpreted as an unacceptable departure from professional or ethical norms. Support for mandates was thus accompanied by a narrowing – implicit or explicit – of what counted as legitimate inquiry, in which critical evaluation of policy outcomes was treated as ideological deviation rather than as a routine component of empirical or ethical assessment. Within this group, positive alignment with institutional policy extended beyond endorsement to objections about the inclusion of questions that invited critical reflection on mandate enforcement or vaccine-related harms. In these responses, compliance was framed as both scientifically justified and ethically required, and questioning the policy was regarded as inappropriate within professional settings.

### Neutral sentiment toward mandates

None of the 80 responses were categorized as neutral. While some responses contained ambiguous or tentative language, each participant ultimately conveyed a clear evaluative stance either in support of, or in opposition to, vaccine mandates – or to the broader institutional and ethical frameworks under which they were imposed. The absence of neutral responses suggests that the mandates were experienced and interpreted by respondents in distinctly evaluative terms.

## Discussion

Our sentiment analysis of 80 respondents from a convenience sample of 189 Alberta healthcare workers found that a clear majority (82.5 %) expressed negative sentiments toward workplace Covid-19 vaccine mandates, while fewer than one fifth (17.5 %) were positive; no responses were neutral, indicating the degree to which the policy was experienced in strongly evaluative terms. Negative responses centered on coercion, ethical conflict, professional exclusion, institutional betrayal, and suppression of dissent. Notably, many vaccinated respondents described compliance under duress, complicating the widespread assumption that vaccine uptake reflects endorsement of vaccination or mandates (see for instance [60]), suggesting instead that uptake may reflect coercion when refusal carries consequences described as too costly to bear.

The most salient theme among entries expressing negative sentiments was moral injury, reported as arising not from isolated clinical decisions but from policy directives embedded in broader structural conditions governing healthcare practice. Among respondents who supported the mandates, by contrast, dissent was often framed as unprofessional, while the survey’s openness to critique was interpreted as bias – an inappropriate move within professional contexts, as if even the exploration of alternatives fell outside the boundaries of acceptable inquiry. These responses align with what Gieryn [61] termed “boundary-work” – the discursive demarcation of legitimate from illegitimate knowledge claims within scientific and professional communities [61]. They also align with broader patterns of dissent marginalization that Martin [62] has documented in scientific controversies, where challenges to dominant positions are often dismissed, misrepresented, or disciplined [62]. In this context, mandate compliance was framed not only as scientifically justified but as ethically non-negotiable, further constraining the space for critical inquiry within professional settings.

It should be noted, however, that while this perspective may reflect intolerance, particularly when directed toward colleagues who experienced exclusion, job loss, and professional displacement, it may also reflect more complex moral dynamics. Under the “state of exception” produced by the Covid policy response [63], and reinforced by highly coordinated public health messaging that framed mandates as both scientifically necessary and ethically non-negotiable, some healthcare workers may have internalized these imperatives as matters of professional conscience. In this context, remaining unvaccinated – or even failing to actively embrace vaccination – could be experienced as a violation of the injunction to “first do no harm”.

This moral urgency, shaped by institutional authority and crisis framing, may be interpreted as groupthink, described by Irving Janis [64] as a mode of collective reasoning in which perceived consensus narrows tolerance for dissent and recasts disagreement as dangerous [64]. It can also be understood through what Callahan [65] and Zola [66] identified as the moralizing function of health language in modern societies, where health becomes a vehicle for social conformity and moral obligation [65], 66]. Under such conditions, dissent suppression can appear professionally and ethically warranted.

These competing yet plausible interpretations highlight the power of institutional messaging to shape the moral landscape within which healthcare workers operated during the Covid era, a messaging prominent in policy-facing expert guidance. A salient example is an Ontario COVID-19 Science Advisory Table brief, which described vaccination as a “health behaviour” and recommended strategies to increase healthcare worker “confidence and uptake”, including tailored messaging, validation of emotions, and recruitment of trusted leaders to help to close the gap between intention and action [67]. Although focused on voluntary vaccination, the brief reflects a broader logic within public health communication: the empirical warrant and ethical legitimacy of a policy objective - in this case, increasing vaccination uptake among healthcare workers - are taken for granted, while barriers to that objective - beliefs, emotions, social networks, and so on - are treated as obstacles to be managed. In doing so, the brief illustrates how healthcare workers’ concerns can be organized primarily as putative “hesitancy”, low confidence, mistrust, defective morality, or deviant political ideology, rather than as empirical, ethical, or professional objections requiring independent assessment.

Our findings can be situated alongside recent studies of healthcare workers who declined, questioned, or resisted Covid-19 vaccination. This literature identifies several patterns that overlap with our findings, including concerns about safety and effectiveness; mistrust of authorities; perceived coercion and stigma; employment consequences; workplace division; and loss of professional standing [2], [18], [19], [20]. The present study documents similar experiences; however, unlike much of the literature, we interpret them not through the language of “vaccine hesitancy”, “misinformation”, or “anti-vaccination ideology” – as “problems” to be explained, managed, or overcome. Rather, drawing on Bacchi’s approach to “problematize” dominant problem representations [68] and on Martin’s framework to understand dissent suppression [62], we treat respondents’ accounts as evidence of both a frame that must itself be problematized and of the ways mandate policy was experienced, morally evaluated, and contested by healthcare workers themselves.

## Limitations and strengths

This study has limitations, some common to qualitative inquiry and others specific to the contested nature of Covid-19 vaccine mandates. First, the data were drawn from a non-probability, convenience snowball sample, which limits statistical generalizability. However, qualitative research does not aim for statistical generalization but for transferability – the extent to which findings resonate with or inform other contexts – which we sought to support through thick description and contextual detail [69]. It is also worth noting that generalizability limitations are not unique to qualitative research. Quantitative studies frequently rely on non-probability samples and on classificatory schemes and variables that are themselves institutionally constructed. As Bowker and Star have shown, categories and variables are not neutral discoveries, but analytic decisions that embed particular values, assumptions, and exclusions, even within rigorous statistical research [70].

Second, although recruitment was open to healthcare workers of all vaccination statuses, the sample was skewed toward vaccine-skeptic or unvaccinated participants, many of whom experienced direct punitive consequences from the mandates. As a result, their perspectives should not be interpreted as estimating the distribution of sentiment among healthcare workers in Alberta. However, these accounts provide critical insight into experiences that have often been marginalized or excluded from dominant narratives, and counterbalance patterns in the literature where compliant, vaccinated healthcare workers are overrepresented. An instance of this overrepresentation is a systematic review of healthcare worker attitudes toward mandatory vaccination, in which authors themselves admitted to potential bias, because most studies selected for review involved predominantly vaccinated workers [71] – a predictable limitation in a context of widespread vaccination-or-termination policies. In this respect, our study prioritizes analytic attention to ethically consequential harms over proportional representation of majority opinion.

Compounding this imbalance, we note that research on dissenting perspectives when scientific matters are involved is inherently difficult. As scholars have noted, experiences that diverge from official narratives are often dismissed or mischaracterized, [62], [72], [73], [74]. The Covid-19 period is no exception. As we have documented, dissent has been strongly suppressed, stigmatized, and marginalized within mainstream discourse, including in expert circles [56], [57], [58], [75]. This context likely contributed to the willingness of some healthcare workers to participate in the study, given the limited availability of venues in which their perspectives could be voiced.

Finally, it could be argued that this study is of limited utility given its focus on Alberta, a province of approximately five million people within a broader national and global context. However, while across Canadian provinces vaccine mandates for healthcare workers followed similar timelines and logics – even as their reception and consequences varied – across these jurisdictions we documented similar experiences of coercion, professional ostracism, and unreported impacts on patient care [54], 55], 76], 77]. What emerges is a transferable pattern not merely of contested bioethics in the healthcare workplace, but of mandates implemented through what has been described as “democratic coercion”: enforcement practices typically associated with authoritarian contexts but rendered acceptable within democratic regimes through appeals to public health emergencies [78]. While Canada is often portrayed as a model of democratic governance and evidence-informed policy, these cases suggest that, in moments of perceived crisis, institutional force may displace deliberative consensus. Alberta healthcare workers across vaccination statuses contribute to this broader picture by articulating dissenting experiences that illuminate the emotional and ethical terrain they navigated under extraordinary pressure, a pattern not uncommon in public health, in which obedience to authority is often projected as standing for professionalism and ethical conduct [79].

This study also has notable strengths. First, while high vaccination uptake among healthcare workers is often interpreted as endorsement of vaccine mandates, our findings – together with national data – complicate the assumption that compliance indicates consent. A 2023 national survey conducted by Ipsos for the Public Health Agency of Canada found that the requirement to maintain one’s job or continue employment was among the most commonly cited reasons for vaccination, reported by 53 % of healthcare professionals, 46 % of allied health workers, and 47 % of auxiliary health workers; 11 % across the three groups identified the employment requirement as their sole reason for vaccination. The report further estimated that, without mandates, as many as 32 % of healthcare professionals and allied health workers and 45 % of auxiliary health workers might not have chosen vaccination [80]. These findings make clear that negative employment consequences played a substantial role in driving uptake, that high uptake cannot be treated as evidence of unconstrained agreement or professional consensus, and that vaccination under duress can be rendered invisible in institutional data that equates uptake with acceptance.

Second, the study complicates claims that Covid-19 vaccination mandates in healthcare workplaces were, or remain, necessary to protect patients or preserve healthcare capacity. Such claims continue to appear in segments of the academic literature that characterize dissent from public health interventions as risk denial, misinformation, or anti-science sentiment (see for instance [81]), or frame the ethical tension within mandated vaccination as one between individual autonomy on the one hand, and patient protection or the public good on the other [21]. These positions often assume what requires demonstration: that employment-based vaccination mandates for healthcare workers were necessary, proportionate, and evidence-informed. While it does not follow that healthcare workers’ autonomy is absolute, or that professional obligations to patients and communities are secondary, what does follow is that appeals to collective welfare cannot substitute for evidence that a restrictive employment policy was necessary, proportionate, and less harmful than available alternatives.

As outlined in the Introduction, however, the mandate rationale depended on empirical assumptions about infection risk, transmission prevention, naturally acquired immunity, vaccine effectiveness, and vaccine safety that were contested at the time and have remained the subject of debate. These empirical matters have tangible implications not only for the well-being of healthcare workers, but also for the sustainability of health systems; failure to address them is reflected in the continued use of vaccination requirements as conditions of employment in some Canadian healthcare institutions [82], 83], even where the relevant doses were administered years earlier and are unlikely to offer any meaningful protection – to healthcare workers or to patients [84], 85].

Third, perhaps the most significant contribution of this study is that, to our knowledge, it is the first to position moral injury as a critical lens for understanding the long-term ethical consequences of public health policy. Existing literature on moral injury among healthcare workers during Covid-19 has primarily focused on frontline trauma, triage, patient mortality, resource scarcity, understaffing, redeployment, lack of personal protective equipment, and institutional failure to support workers under crisis conditions [45], 86], 87], while continuing to centre the “problem” largely around the healthcare worker.

Our reading of respondents’ accounts suggests an additional source of moral injury – one that only becomes apparent if the policy itself is subject to critical scrutiny: this source is the systematic enforcement of policies experienced as ethically coercive. In this light, accounts from healthcare workers indicate moral dislocation, while also representing critiques of the policies. Our findings therefore extend the literature by showing that moral injury can also arise from employment-based policy enforcement when healthcare workers perceive themselves as compelled to comply with, enact, or remain silent about a policy they regard as inconsistent with informed consent, professional ethics, patient care, or evidentiary integrity.

At the same time, we acknowledge that the concept of moral injury, while useful, carries certain risks. Once incorporated into the health sciences lexicon, moral injury may become medicalized – reframed primarily as a form of psychological or psychiatric dysfunction rather than as an expression of systemic ethical rupture [66]. Such a shift risks relocating responsibility from structural and political conditions to individual resilience. In our view, responding to moral injury among healthcare workers cannot be limited to clinical intervention, but would also involve a structural rethinking of public health ethics that prioritizes transparency, proportionality, inclusivity, and respect for fundamental bioethical principles – most critically, informed consent [88], 89]. The harms identified in this analysis are not merely personal but systemic, and meaningful response must therefore engage structural and collective dimensions.

## Conclusions

This study examined the lived experiences of Alberta healthcare workers subject to workplace Covid-19 vaccine mandates and found that compliance should not be equated with agreement, consent, or ethical alignment. By foregrounding perspectives across vaccination statuses, our findings show that high vaccine uptake can obscure the coercive and morally fraught conditions under which such uptake was achieved. They also suggest that moral injury in this context was not merely an individual psychological response, but a structurally induced ethical harm arising from governance practices that constrained professional judgment, narrowed acceptable dissent, and placed informed consent, patient care, and employment security in tension. Treating dissent as an empirical object rather than a deficit to be corrected, this study offers a sociologically informed analysis of health governance, professional autonomy, and moral regulation under crisis conditions. Preventing moral injury in the health sector requires more than post hoc support for distressed workers; it requires policy design that preserves ethical and institutional integrity before harm is produced.
